# QuickStats

**Published:** 2015-01-23

**Authors:** FROM THE NATIONAL CENTER FOR HEALTH STATISTICS

**Figure:**
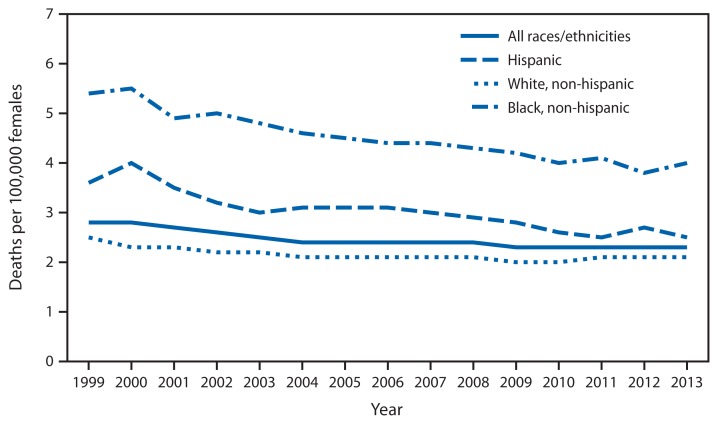
Death Rates* for Cervical Cancer^†^ — National Vital Statistics System, United States, 1999–2013 * Age-adjusted rates (deaths per 100,000) based on the 2000 U.S. standard population. Populations used for computing death rates for 2011–2013 are postcensal estimates based on the 2010 census, estimated as of July 1, 2013. Rates for census years are based on populations enumerated in the corresponding censuses. Rates for noncensus years before 2010 are revised using updated intercensal population estimates and might differ from rates previously published. ^†^ Malignant neoplasm of cervix uteri (*International Classification of Diseases, 10th Revision* [ICD-10] code C53) as the underlying cause of death includes the following ICD-10 codes: endocervix (C53.0), exocervix (C53.1), overlapping lesion of cervix uteri (C53.8), and cervix uteri, unspecified (C53.9).

In 2013, the age-adjusted cervical cancer death rate was 2.3 per 100,000. The rate for non-Hispanic black females was nearly double the rate for non-Hispanic white females (4.0 compared to 2.1) and 1.6 higher than the rate of 2.5 for Hispanic females. From 1999 to 2013, cervical cancer death rates have decreased 31% for Hispanic females, 26% for non-Hispanic black females, and 16% for non-Hispanic white females.

**Source:** National Vital Statistics System. Mortality public use data files, 2013. Available at http://www.cdc.gov/nchs/data_access/vitalstatsonline.htm.

**Reported by:** Betzaida Tejada-Vera, MS, fsz2@cdc.gov, 301-458-4231.

